# Characterization of Coffee Silver Skin as Potential Food-Safe Ingredient

**DOI:** 10.3390/foods10061367

**Published:** 2021-06-13

**Authors:** Maria Martuscelli, Luigi Esposito, Carla Daniela Di Mattia, Antonella Ricci, Dino Mastrocola

**Affiliations:** Faculty of Bioscience and Technology for Food, Agriculture and Environment, University of Teramo, Via R. Balzarini 1, 64100 Teramo, Italy; lesposito2@unite.it (L.E.); cdimattia@unite.it (C.D.D.M.); aricci@unite.it (A.R.); dmastrocola@unite.it (D.M.)

**Keywords:** coffee by-products, dietary fiber, color, minerals, bioactive compounds

## Abstract

By-products from the coffee industry are produced in large amounts each year. Among other wastes, coffee silver skin (CSS) is highly available and more stable due to its lower content of water. This research aimed to characterize coffee silver skin composition and evidence its potentiality for use as a food-safe ingredient in new formulations. Results showed an average total dietary fiber content of 50% but with a higher ratio for insoluble than soluble fiber. A high content of total phenolic compounds, chlorogenic acid, caffeine, and caffeic acid was found and correlated with the high measured antioxidant capacity. Moreover, minerals (e.g., calcium, magnesium, phosphorous, potassium, copper, iron, manganese) important for human wellbeing were found at a high level in CSS, while toxic minerals (e.g., nickel) were found at low levels. In conclusion, coffee silver skin could have an advantageous role for the recovery of valuable compounds and as a potential food-safe ingredient.

## 1. Introduction

A real and effective solution to ensure access to safe and secure food is to limit food waste at the industry level and in home consumption [1]. Whatever the supply chain, a significant number of by-products, that are difficult to reuse and potentially responsible for environmental pollution, are produced [2,3]. Furthermore, the valorization and reuse of food by-products fulfills sustainable development goals, specifically the 12th (responsible consumption and production) and the 13th (climate action) goals, established by the Food and Agriculture Organization (FAO agenda 2030).

Coffee silver skin (CSS) is a thin layer that covers coffee seeds inside the coffee “cherries” of the genus *Coffea* of Rubiaceae plants (*Coffea arabica* and *Coffea canephora*). CSS is the unique by-product discarded after the roasting process; coffee production gives rise to other by-products, such as coffee pulp and coffee husks, before the roasting step and spent coffee grounds after coffee preparation [4]. Since coffee is one of the most consumed beverages worldwide and one of the most traded items globally, its industry generates a lot of residues [5,6]. CSS represents 4.2% (*w*/*w*) of seed total weight, with values of 1 ton of CSS for every 120 tons of roasted coffee [7]. Despite being a less abundant coffee by-product, the availability of CSS justifies the interest in its recovery and use in several applications in different sectors, not just for the food industry. CSS has been proposed as a material for renewable energy sources, as an electrode material, and as a biofuel source [7].

Due to its content of valuable compounds like fibers, sugars, and polyphenols, it would be interesting and useful to exploit it as an innovative and functional food ingredient [8]. Costa and co-authors reported that different cultivars (*C. arabica* and *C. canephora*) have comparable phenolic profiles and compositional traits, and they were successfully utilized as food antioxidants and fiber integrators [9].

Coffee’s provenience greatly influences its composition; thus, it is difficult to exactly determine it in each variety cultivated. This substrate contains a lot of dietary fiber (up to 55%), including both insoluble (≈45%) and soluble (≈10%) fractions [10]. Cellulose is present at a high level (≈60%), which is helpful for the growth of many types of bacteria and molds to produce enzymes like α-amylase and fructooligosaccharides. According to Esquivel and Jiménez [11], CSS can be easily incorporated into preparations due to its low content in lipids and carbohydrates. Recently, some authors found that CSS is also a good source of relevant amino acids [12].

Some studies explored the possibility to use CSS as a food ingredient in several matrices, despite little information about levels of ochratoxin A and other contaminants being available. In bread, CSS alkalinization was proposed to increase water-holding capacity and the content of fibers [13]. More recently, CSS was added to yogurt [14], to biscuits as a source of dietary fibers and prebiotics [15], and was also blended with instant coffee for new coffee-based beverages [16]. Due to its content of melanoidins, high molecular weight compounds mainly constituting polysaccharides, proteins, and chlorogenic acid molecules [17], CSS can find application as a food dye. Moreover, melanoidin contributes to the antioxidant activity of CSS, showing anti-inflammatory effects on different organs [18].

For marketing within the European Union (EU), it is very important to define whether products, like CSS, need to obtain approval as an ingredient for novel food [4], with special reference to its legal status within the EU and potential options for producers to obtain approval according to the Novel Food Regulation (EU) No 2015/2283 and the two implementing regulations (EU) No. 2017/2468 and No. 2017/2469) [19,20].

In this scenario, the present research investigated the chemical and physical properties of CSS; it also found the volatile profile and mineral composition to enhance its global potentiality to be considered as an advantageous ingredient in newly formulated foods.

## 2. Materials and Methods

### 2.1. Coffee Silver Skin

Coffee silver skin (CSS) was received from the toasting plant Marcafè Torrefazione Adriatica s.p.a. (Giulianova, Italy). After 10 cycles of roasting (10 × 240 kg of roasted coffee), 3.3 kg of CSS were recovered. CSS used for this experiment was a blend of 5 arabica varieties (*C. arabica*) (India Arabica, India Cherry, Vietnam, India Mysore, and India Caracolito) and 5 robusta varieties (*C. canephora*) (India Parchment, Santos, Uganda CRV 18, Uganda CRV 17, Togo). CSS was ground at 10,200 rpm for 1 min (Bimby^®^, mod. TM 31, Wuppertal, Germany) until reaching a particle size of 250–125 µm.

Then, physico-chemical, physical, and chemical analyses were carried out. CSS was kept frozen at −20 °C until analysis.

### 2.2. Physico-Chemical, Color, and Compositional Analyses

Water activity values (a_w_) were obtained with the Aqualab 4 TE kit (Court Pullman, WA, USA). Values of pH were taken with a pH meter (model 3510, Jenway, Stone, UK) in triplicate and expressed with their standard deviation.

Colorimetric parameters were obtained with a CR-5 colorimeter (spectrally based, Konica Minolta, Japan) with a D_65_ light source and observer 10°. Samples were measured in triplicate for a total of 9 measurements. To better define the final color observed, the saturation index (chroma, C^*^) was calculated according to Formula (1):

C^*^ = (a^*2^ + b^*2^)^1/2^ (1)

Proximate analysis of moisture, proteins, lipids, and ash was carried out with different protocols. Moisture, ash, and proteins were determined following the Association of Official Analytical Chemists procedure [21], as follows. Moisture content was determined by drying about 1 g of sample to a constant weight. Ash was determined by the calcination of 1 g of sample in a furnace at 525 ± 25 °C until a constant weight was reached. Total nitrogen content (TN, % *w*/*w*) was determined using the standard Kjeldahl copper catalyst method and the protein content was calculated by multiplying TNx6.25.

Total lipids were measured using a modification of the chloroform to methanol procedure described by Folch et al. [22]. Total dietary fiber, insoluble fiber content, and soluble fiber content were determined with a Megazyme Integrated Total Dietary Fiber Assay Kit that was accepted as AOAC Method 2017.16 in 2018 and is the only method consistent with the CODEX Alimentarius definition of dietary fiber [21].

Briefly, 1 g dried food sample (duplicate) was subjected to sequential enzymatic digestion by heat-stable α-amylase, protease, and amyloglucosidase. Insoluble dietary fiber (IDF) was filtered, and then the residue was washed with warm distilled water. The combined solution of filtrate and water was precipitated with 4 volumes of 95% ethanol (EtOH) for soluble dietary fiber (SDF) determination. The precipitate was then filtered and dried. Both SDF and IDF residues were corrected for protein, ash, and blank, for the final calculation of SDF and IDF values. 

#### 2.2.1. Acrylamide Content

To determine acrylamide in CSS, an analysis was performed by the external laboratory Chelab s.r.l. (Accredia N° 0051L), by means of HPLC/MS (triple quadrupole) according to an internal procedure. The limit of quantification was 50 μg kg^−1^ (as CSS is a dried vegetable matrix); the limit of detectability was 15 μg kg^−1^ (as CSS is a dried vegetable matrix).

#### 2.2.2. Water-Holding and Oil-Holding Capacity (WHC, OHC)

Water- and oil-holding capacities (WHC and OHC) were measured according to the procedure of Ballesteros et al. [23], with some modifications. Our results come from 6 independent measurements.

For WHC, 1 g of CSS was placed in 30 mL plastic tubes, with 10 mL of distilled water, then vortexed for 1 min and left in the dark for 24 h. After this time, samples were centrifuged for 20 min at 3000 rpm to separate the unabsorbed water from the CSS. The pellet obtained was weighed and the variation was expressed as grams (g) of absorbed water.

The same method was followed to determine the OHC by using 0.5 g of CSS and 5 g of sunflower oil.

### 2.3. Polyphenol Extraction, Total Polyphenol Content (TPC), and Phenolic Profile Determination in CSS

The extraction of the phenolic fractions from CSS samples was carried out by using different solvent mixtures: deionized water and methanolic aqueous solutions at two methanol concentrations (60:40 and 70:30 *v*:*v*). The procedure was as follows, 1 g of sample was added to 10 mL of the solvent; the mixture was vortexed for 1 min and then centrifuged (4000 rpm for 10 min). The supernatant was recovered and filtered through 0.45 μm nylon filters. For the extraction of the bound phenolic compounds, the insoluble residue from the extraction carried out with the 70% methanolic solution was recovered and re-extracted with 10 mL of a methanol:water:chloridric acid solution (70:29:1), vortexed for 1 min, stirred at room temperature for 2 h, and then centrifuged and filtered by using the same conditions as before. The extracted polyphenols were then stored at −32 °C until further analysis. These extracts were used for the evaluation of total polyphenol content and radical-scavenging activity. The analysis of the phenolic pattern was carried out on the 70% methanolic extract. The total polyphenol content (TPC) was determined following a modified procedure from Di Mattia et al. [24]. Water was added to 0.1 mL of sample extracts up to a volume of 5 mL; 500 μL of Folin–Ciocalteu reagent was then added. After 3 min, 1.5 mL of a 25% (*w*/*v*) Na_2_CO_3_ solution was added, followed by deionized water up to a 10 mL final volume. Solutions were maintained at room temperature away from light sources for 60 min. Absorbance was measured at 765 nm using a Perkin Elmer Lambda Bio 20 spectrophotometer. Gallic acid standard solutions (Fluka, Buchs, CH, Switzerland) were used for calibration. Results were expressed as mg of gallic acid equivalents (GAE) per g of sample.

The identification and further quantification of phenolic acids were conducted by means of HPLC-DAD analysis (mod. HPLC: AGILENT series 1200 Agilent Technologies, Milan, Italy), in accordance with the method suggested by Horžić et al. [25]. A reverse-phase C18 column (5 µ 18 100A 250 × 4.6 mm, Phenomenex Kinetex, Bologna, Italy) was used. The solvent phases utilized were water and formic acid 5% (A), and methanol (B). The flux was fixed at 1 mL/min. Table 1 reports the gradient used for the analysis. Data for chlorogenic and caffeic acid were spectrophotometrically read at 280 nm while caffeine was read at 330 nm. The quantification was determined using calibration curves in a range from 6.25 to 100 mg/L.

### 2.4. Melanoidin Determination of CSS

The melanoidin content was determined by spectrophotometric analysis by reading the absorption value at a wavelength of 345 nm [26] on the dialyzed fraction of the aqueous extract obtained as described in Section 2.3. Before melanoidin analysis, 5 mL of the aqueous extracts were dialyzed using cellulose dialysis tubing (MWCO: ~12,400 Da) (Sigma Aldrich Italy, Milan, Italy) placed in a glass vessel with l L of deionized water and maintained under stirring for 12 h. This procedure was repeated after refreshing the deionized water. The volume of the CSS aqueous extract which remained in the dialysis tube was diluted to a known volume with deionized water.

### 2.5. Radical-Scavenging Activity (TEAC) of CSS

The radical-scavenging activity was measured by the 2,2′-azino-nis (3-ethylbenzthiazoline-6-sulfuric acid (ABTS) radical cation discoloration assay [27]. The ABTS radical stock solution was prepared by dissolving the ABTS in water to a 7 mM concentration and by reacting this solution with 2.45 mM of potassium persulfate; the mixture was then left in the dark at room temperature for 12–16 h before use. The ABTS+• stock solution was diluted in water to an Abs of 0.70 ± 0.02 for the analysis and the reaction was started by the addition of 30 µL of CSS extract to 2.97 mL of ABTS+• radical solution.

The bleaching rate of ABTS+• in the presence of the sample was monitored at 25 °C at 734 nm using a Perkin Elmer (Boston, MA, USA) Lambda Bio 20 spectrophotometer, and the discoloration after 5 min was used for the calculation of the inhibition percentage.

Radical-scavenging activity was expressed as mol Trolox equivalents antioxidant capacity (TEAC-µmol of Trolox equivalents per g of sample) and calculated by the ratio of the correlation coefficient of the dose–response curve of the sample and the correlation coefficient of the dose–response curve of Trolox, the standard compound.

### 2.6. Mineral Determination of CSS

A microwave digestor (mod. Speedwave 2) (Berghof groups, Germany) coupled with inductively coupled plasma for the reading of the results (ICP-OES, mod. Avio200) (Perkin Elmer, Boston, MA, USA) were used, in accordance with UNI EN 13657:2002 [28].

Mineral determination followed the instructions of the manufacturer. Samples were weighed and added to 3 mL nitric acid and 3 mL oxygen peroxide left for 20 min at room temperature to start the digestion. After this, samples were submitted to the specific ramp in line with the Berghof manual procedure for coffee samples. Results are expressed as the average of triplicate analysis.

Once digested, samples were left at room temperature overnight. When cold, they were filtered with filter paper. A solution of 5% of nitric acid was used to clean vessels and to suspend the analytes obtained. This solution was brought to the final volume of 50 mL by adding nitric acid 5%.

The reading of samples was done by developing an internal method in ICP software (Syngistix) by selecting defined parameters and making a calibration curve. For this, nitric acid was used as a blank and washing solution. Certified standard solutions were used as standards containing all analytes traced at a concentration of 100 mg/L.

### 2.7. Volatile Compounds in CSS

To investigate the volatile compounds, profile GC-MS analysis was performed (Perkin Elmer Gas Chromatograph Clarus 580, Mass Spectrometer Perkin Elmer Clarus SQ 8 S Waltham, MA, USA). Approximately 2 g CSS was put in glassy vials of 20 mL capacity (Perkin Elmer), tightly closed, and stocked at −40 °C, assuring the highest headspace. For the GC-MS analysis, the method used was taken from Qi et al. [29] with some modifications

Briefly, volatiles from CSS were extracted with a headspace solid phase microextraction fiber (SPME 65 μm polydimethylsiloxane/divinylbenzene -PDMS/DVB-; Supelco, Bellofonte, USA) and collected for 30 min at 40 °C, then inserted into the GC injector and desorbed for 3 min at 250 °C. Volatile compounds were separated on a ZB-SemiVolatiles capillary GC column (30 m length × 0.25 mm internal diameter × 0.25 μm film thickness, Phenomenex, Torrance, CA, USA).

The oven temperature was maintained for 3 min at 40 °C, increased at 3 °C/min to 70 °C, then at 5 °C/min to 180 °C, then at 10 °C/min to 260 °C, and maintained for 5 min at 260 °C.

Helium was the carrier gas with a constant flow of 1mL/min.

The mass-selective detector was operated in electron impact mode (70 eV) and full scan mode (35–500 *m/z* range). The identification was performed using the National Institute of Standards and Technology mass spectral library (NIST Mass Spectral library, Search Program version 2.0, National Institute of Standards and Technology, U.S. Department of Commerce, Gaithersburg, MD, USA).

### 2.8. Statistical Analysis

All determinations were done in triplicate, except where indicated otherwise. Means and relative standard deviations were calculated. The statistical differences of the results on the content of phenolic compounds and on the antiradical activity between the soluble and bound fractions were determined by a *t*-test between means; the analyses were carried out by XLSTAT software (Addinsoft SARL, New York, NY, USA).

## 3. Results

### 3.1. pH, a_W_, and Color of CSS

pH measurements gave values of 5.34 ± 0.018 (Table 2). No bibliographical references have been found to compare our results; however, other authors have reported pH levels of 4.9–5.6 for coffee beverages, and they considered these pH values suitable for the activity of biomolecules (e.g., polyphenols) [30]. As already mentioned, CSS has a coarse structure and dusty appearance which make it suitable for adding to food formulations and easily stored. Our values confirm this characteristic even if no reference is available to compare these values.

Very low water activity values were found (0.319 ± 0.00), which is important evidence for the low availability of water for degradation reactions in CSS [31]; consequently, its stability depends on the environmental humidity conditions in which the CSS is stored, which must be such as to prevent water absorption and provide a prolonged shelf life to CSS.

Color, L*, a*, and b* parameters were like those reported in the literature [13]. The color mainly depends on the interaction of constitutive polyphenols and anthocyanins, which are involved in oxidation and polymerization reactions during the coffee roasting step [32]. Moreover, roasting processes cause degradation of proteins, and Maillard reactions occur, so melanoidins also contribute to the characteristic color of CSS.

### 3.2. Characteristics of CSS Composition

Table 2 reports data on the composition of CSS. As previously mentioned, CSS is a low-moisture ingredient due to the roasting step that also lowers the a_W_ value. Our results are close to data from Costa et al. [9] (4.76 ± 0.10 g/100 g). Gocmen et al. [33] substituted wheat flour with different concentrations of CSS (2.5, 5.0, and 7.5%) for cookie production, observing darker products. Similarly, Garcia De Serna et al. [15] used CSS as a substitute for sucrose in combination with stevia at concentrations of 1.33 and 3.33 g, obtaining darker biscuits. Finally, in sponge cakes, Ateş and Elmacı [34,35], used concentrations of 20, 25, and 30% as fat replacers, observing a decrease in L* and b* values of the crumb and an increase in a* value. They also added CSS as a substitution for flour [35], observing a higher index for crumb darkness and redness and an increase in fiber content, concluding that no strong difference could be observed with additions up to 30%.

Fiber content in by-products gave good results, confirming what is stated in the literature (Table 2). CSS has an average TDF content of 50% but a high ratio between IDF and SDF. Costa et al. [9] also found a TDF% content of 56.4 ± 0.70, IDF level of 49.1 ± 0.44, and SDF of 7.30 ± 0.07. Iriondo-De Hond et al. [10] found higher levels; their results were 67.7 ± 1.6 (TDF%) for arabica varieties (*C. arabica*), IDF content 64 ± 1.6, and SDF 3.7 ± 0.0. In comparison with these results, the study presented a major SDF content of 7.86%, although other studies gave SDF% values of around 8.8% for arabica varieties (*C. arabica*) [36]. As for other compounds, this variability depends on roasting conditions, geographical provenience, and the varieties analyzed. Despite these differences, the consumption of CSS can improve the daily intake of dietary fibers, known for their beneficial role in lowering the risk of cardiovascular diseases, obesity, type 2 diabetes, chronic constipation, and other unhealthy conditions [37,38]. Moreover, as highlighted by [39], the consumption of foods rich in dietary fibers are often good reservoirs of phenolic compounds which have antioxidant and anti-inflammatory effects. According to [40], the recommended dietary reference intake (DRI) daily allowance in men aged 19–50 years is 38 g/day and in women 25 g/day, and for men aged > 51 it is 31 g/day and in women aged > 51 it is 21 g/day, although the average consumption among Americans is about 15 g/day. The quantity of dietary fibers in vegetable foods varies a lot, and the ratio of IDF and SDF differs. Cereals and some fruits, vegetables, and nuts are considered good sources of dietary fibers. For wheat (*Triticum aestivum* L.), the common values are 11.6–17.0 g TDF, of which 10.2–14.7 g are IDF, and 1.4–2.3 g are SDF; for oats (*Avena sativa* L.), the values are 10.3 g TDF, of which 6.5 g are IDF, and 3.8 g are SDF [41]. In apples (*Malus domesticus*), these concentrations were found to be 4.4 g TDF, 3.3 g IDF, and 1.1 g SDF; oranges (*Citrus sinensis* L.) have 3.4 g TDF, 1.4 g SDF, and 2.0 g IDF [42].

A content of 720 ± 110 μg kg^−1^ of acrylamide was found. Recent limits established by the European Commission are 400 μg kg^−1^ for roast coffee, 850 μg kg^−1^ for instant (soluble) coffee, and 500–4000 μg kg^−1^ for coffee substitutes, reported in the Commission Regulation (EU) 2017/2158 of 20 November 2017 [43]. These values cannot be directly compared, as the use of CSS is not for coffee substitution. Instead, other acrylamide limit values must be considered to establish, depending on the final products, the amount of CSS as a functional component that can be added. The content we found was higher than 489 μg kg^−1^, which was the value reported for coffee silver skin aqueous extracts [10]. However, the coffee silver skin analyzed in that study only came from the arabica variety (*C. arabica*) of coffee, while our sample included a contribution of 50% from the robusta variety (*C. canephora*), for which a higher content of acrylamide produced upon roasting has been described in the literature [44].

### 3.3. Water-Holding and Oil-Holding Capacity

Water- and oil-holding capacities are important indexes to express the capability of an ingredient to retain water or oil and give a formulation more moisture and softness, influencing the final texture and palatability of a product. CSS has already been tested in bakery products and bread, acting as a moisturizing ingredient and a fat replacer [13,15,34].

In some of these studies, CSS was alkalizated to enhance its functional properties, but even when used raw, it had remarkable abilities as an adsorbent material.

Our results (Table 2) are comparable with those published by other authors [13,24] who have shown how the WHC is affected by the fiber content and type, which was mainly soluble, while differences for the OHC index can be imputable with the particle size of the investigated samples and the different oils used (corn oil vs. sunflower oil).

In their studies, Ateş and Elmacı [34,35] obtained higher results than us for OHC, (4.82 ± 0.23) but they are comparable, as they also used sunflower oil for the analysis. From their work, they determined that water-treated CSS can be a useful fat replacer in sponge cake and a flour replacer.

Previously, Behrouzian et al. [45] performed an optimization study for the utilization of CSS in food, establishing the most suitable conditions for an alkaline hydrogen peroxide treatment. With their attempts, they showed the effect of temperature and pH conditions on both WHC and OHC and also on cellulose extraction.

Belmiro et al. [46] suggested that matrices that are abundant in dietary fiber are affected by their molecular size, degree of branching, intermolecular aggregation, and water-binding sites and, consequently, their technological properties could be modified by physical or chemical treatments. This may induce a reduction in particle size and/or enhancement of particle porosity, and permeability and diffusion of water or oil into the fiber. These authors have shown that the WHC and OHC of coffee co-products are enhanced by higher pressures (100 MPa), by a minimum concentration of acetic anhydride (10%), or by enzymatic treatments (at least 5 U of cellulase for 30 min); in all tested methods, changes were attributed to the disruption or disordering of the dietary fiber structure and to the formation of aggregates. However, the same authors observed a significant negative effect of those technological treatments on the antioxidant status of coffee co-products [47].

### 3.4. Polyphenols, Melanoidin, and Radical-Scavenging Activity

The extraction of the phenolic fraction was preliminarily carried out by using deionized water and two solutions containing 60% and 70% (*v*:*v*) of methanol, respectively, in order to explore the extractive capability of solvent mixtures characterized by different polarities, widely used in the literature for vegetables [48].

Among the mixtures, the 70% methanol solution allowed the highest yield of the soluble fraction as evaluated by the content in total phenolic compounds (TPCs) and, for this reason, it was chosen for further investigations on the radical-scavenging activity as well as on the phenolic pattern. Moreover, as phenolic compounds can also occur in the bound form by either ester or ether bonds with sugars, alcohols, and other biomolecules from cell walls, the insoluble residue recovered from the first step was submitted to further extraction under acidified conditions. Ҫelik and Gocmen [49] have recently reviewed all the factors interacting with the availability and extraction of soluble and bound phenols, remarking on the dynamic balance of this system that can change according to the conditions of the medium. The results on the content of phenolic compounds and antiradical activity of the sample, along with the content in melanoidins, caffeine, and chlorogenic and caffeic acids, are reported in Table 3.

The TPC of the soluble fraction was significantly higher with respect to the bound fraction. Due to the differences in the solvent mixtures used, procedures adopted, and molecules used as reference compounds for the assays, the comparison of TPC results with others found in literature was very difficult; however, it can be generally affirmed that larger amounts were found with respect to Gemechu [8], whilst lower values were observed when the work of other authors was considered [36,49,50,51,52].

Besides the factors previously cited, such a discrepancy can also depend on the geographical provenience of CSS and the roasting process; however, a reduced content of these molecules can positively affect bitterness, astringency, and the cut grass smell sensations that are developed with cooking processes. As far as the radical-scavenging activity is concerned, the soluble and bound fractions differed significantly in the TEAC values, with the free fraction showing the highest value. This may be primarily related to the TPC values but maybe also a consequence of the different compositions in the patterns of phenolic compounds of the two extracts, with the soluble one being richer in molecules with higher antiradical activity with respect to the bound fraction. In general, the antiradical activity was comparable to results reported in the work by Borrelli et al. [53].

**Figure 1:**
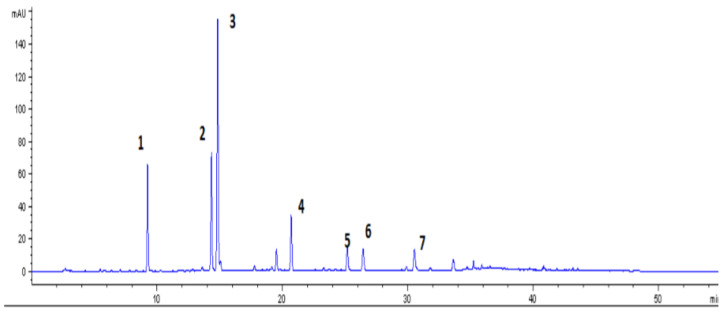
HPLC profile of coffee silver skin at 330 nm of phenolic acids in extract (MetOH:H_2_O 70:30). Identified peaks are: caftaric acid (**1**), chlorogenic acid (**2**), caffeic acid (**3**), ferulic acid (**4**), chlorogenic acid isomers (**5**–**7**).

To gain a more in-depth understanding of the phenolic composition of CSS and to quantify caffein content, the extracts were submitted to HPLC analysis (Figure 1) and in Table 3 the contents of the most representative phenolic acids—chlorogenic and caffeic acids—are reported. Indeed, the major phenolic compounds in CSS are chlorogenic acids, which are esters of cinnamic acids with quinic acid; several isomers have been detected in CSS. However, 5-O-caffeoylquinic acid (5-CQA) was found to be the main one [54].

In the soluble and bound fractions, similar contents of caffeic and chlorogenic acids were found; generally, significantly higher contents were found in the soluble fraction. The amounts of chlorogenic acid found in the present work are generally in agreement with other works, in which contents of 0.12mg/g and 0.46 mg/g were found in CSS samples from India and Uganda, respectively [54].

The same HPLC method allowed the quantification of caffeine, which was found only in the soluble fraction at 17.45 ± 1.2 mg g^−1^, a value which agrees with those found by Bessada et al. [2,54], as well as by Costa et al. [9]. Caffeine was not detected in the bound fraction, likely because, in the samples under investigation, it was not bound to other compounds and, under the experimental conditions used, it was thus possible to achieve a complete extraction with the first step.

Aqueous extracts were dialyzed and used to evaluate the melanoidin fraction; the absorbance at 345 nm had a value of 0.71 ± 0.01; Mésias and De Andrade [55] reported values of 4.5 g/100 g. Melanoidin content and the molecular weight are strictly dependent on the roasting process and temperature range at which coffee beans are roasted, as well as on the number of precursors available for the Maillard reaction.

CSS, and coffee by-products generally, mainly have water-soluble melanoidins, and so they were suggested as resources for the formulation of new products to impart antioxidant and antimicrobial functions when producing functional foods [54,55]. Tores de la Cruz et al. [30] suggest specific coffee compounds as being responsible for acidity, as well as chlorogenic acid, citric acid, quinic acid, trigonelline, caffeine, and arabinogalactan [30,32].

### 3.5. Minerals in CSS

Minerals are highly present in CSS [8]. As for fibers and bioactive molecules, minerals are important nutrients for human wellbeing, representing other valuable compounds to recover. We looked for those microelements that are useful for human health, such as calcium, potassium, and iron. We were able to trace out other minerals, such as phosphorous, magnesium, manganese, copper, sodium, zinc, nickel, chromium, and aluminum. The data obtained are shown in Table 4 and were compared with a few references available on mineral contents of coffee silver skin [9,51]. Costa et al. [9] reported CSS values of potassium (4977 ± 151 mg 100 g^−1^), magnesium (2002 ± 72 mg 100 g^−1^), calcium (584 ± 62 mg 100 g^−1^), iron (41.8 ± 2.69 mg 100 g^−1^), and sodium (5.32 ± 0.14 mg 100 g^−1^). Ballesteros et al. [23] reported values of 470.60 ± 13.9 (mg/kg) for aluminum and 1.64 ± 0.34 (mg/kg) for nickel. This study confirms a high content of calcium, potassium, and phosphorus, and good values of iron, copper, and manganese in CSS. For aluminum, our results suggest lower contents with respect to cited data, as is the case for nickel.

An important point is that CSS results to have large amounts of minerals and can be used as an integrator. Soetan et al. [56] remarked on how humans consume large amounts of minerals, with high needs for calcium and iron, used for bone renovation, the correct functioning of red blood cells, and cellular respiration. Magnesium, copper, selenium, zinc, iron, manganese, and molybdenum are important co-factors found in the structure of certain enzymes and are indispensable in numerous biochemical pathways. Conversely, some minerals are not involved in any biochemical pathway and can even be toxic or being used as a processing contamination index; aluminum and nickel are among them [56,57]. Minerals can have different health effects; however, their daily requirement levels are very low (as they are micronutrients), so a small amount of CSS in a newly formulated food could remarkably increase their intake. According to the World Health Organization (WHO), the daily calcium intake, for men and women from 19–50 years old, is fixed at 840 mg daily [58].

By looking at our data, it can be assumed that in 100 g of CSS, there is about 546.5 mg of calcium, so, keeping these values in mind, an addition of 1.5% of CSS to a final amount of 45 g of a new formulated food will give 16.38 mg of calcium (2% daily requirement). In some studies [59], the daily intake of calcium (for the same target population) is lower, tat 700 mg daily. Similarly, for potassium intake, the WHO [60] recommends 3510 mg daily for adults. Following the previous example, 3g of CSS (1.5% for a 45 g final product) provides 65.7 mg of potassium (1.91% of daily requirement). According to these data, potassium can be used as a replacement for salt (NaCl) in newly formulated foods.

The literature agrees that aluminum and nickel mainly come from fertilization, the high use of pesticides, and fungicides [56], which increase their content in the soil. Other causes are geographical and climatic conditions [57,61].

Aluminum and nickel are among the most abundant metals around the globe (they are normally contained in vegetable sources) and their concentration grows steeply due to the human activities of extraction and soil and water acidification [57,61,62]. Recently, the European Food Safety Agency reviewed the use of aluminum sulfates (E 520–523) and sodium aluminium phosphate (E 541) as food additives due to the higher intake in the human diet [63].

The EFSA statement [64] on the evaluation of aluminum oral bioavailability has shown drinking water values close to 0.3%. Often, the bioavailability of aluminum from food and beverages is lower, at about 0.1%.

However, the oral absorption of aluminum from food can vary at least 10-fold depending on the chemical forms present in the intestinal tract. The total burden of aluminum in healthy human subjects has been reported to be approximately 30–50 mg/kg bw. Considering a formulation containing 1.5% CSS, the aluminum intake would be 0.67g for each 100 g of food eaten. It was concluded that these additives are safe for the human diet, with a no observed adverse effect level (NOAEL) of 52 mg Al/kg body weight (bw) per day in rats. Limits for nickel were recently reviewed by the CONTAM group of EFSA [65] Although average intake from water does not represent a risk, some classes of the young population in Europe seem to be more exposed. The tolerable daily intake assumed to represent a risk had a lowest observed adverse effect level of 4.3 μg Ni/kg bw.

### 3.6. Volatile Organic Compounds (VOCs) in CSS

Volatile organic compounds (VOCs) in CSS, from this study, were grouped into seven classes (Table 5). The order of concentrations is nitrogen-containing compounds, pyrazines, aldehydes, alcohols, other compounds, hydrocarbons, and ketones. In coffee, many volatiles come from the roasting process of beans. Although there are few studies about VOCs from CSS, the typical volatiles found in coffee beans can be assumed for CSS.

Recently, for the first time, Angeloni et al. [66] researched the volatiles of CSS. By HS-SPME-GC-MS, they found more than 80 active odorants, with many of them shown in the data reported here. Furthermore, by GC-O/FID and GC × GC-TOF analysis and comparison with coffee beans, they were able to identify some characteristic compounds, such as 1-octen-3-one, 2-phenylethanol, and γ-decalactone, not seen in this study.

Classes of compounds found here are also reported in other studies [67,68,69], although is very difficult to compare different samples because of the roasting parameters, provenience of coffee beans, their composition (also including the number of defective ones), the blending of cultivars chosen, and the selected processing method (wet vs. dry) [68]. Yang et al. [69] showed how the increase or decrease in thirty-seven typical compounds occurred during the roasting process, highly influencing the quality of the final coffee beans and used as quality markers. Specifically, five defects were found (light, scorched, dark, baked, and underdeveloped) and connected to elevated levels of indole, 4-ethyl-2-methoxy phenol, phenol, maltol, and 2,5-dimethylfuran, respectively. Besides these difficulties, Fisk and co-workers [70] listed fifteen “iconic” compounds which influence the coffee aroma. Among them, butanal, 2-methyl-butanal, 3-methyl-pyrazine, 2-ethyl-6-methyl-, pyrazine, and 3-ethyl-2,5-dimethyl were also present in CSS samples from this study (Table 5). A study on pyrolysis products (bio-oil) of coffee silver skin coupled with a two-dimensional (GC × GC/qMS) analysis [71] showed a great variety and complexity of compounds, as follows: N-compounds, phenols, unsaturated aliphatic hydrocarbons (8.28%), saturated hydrocarbons, aromatic hydrocarbons, ketones, esters, carboxylic acids, alcohols, and 10 compounds classified as “other”. Pyrolysis allowed the authors to list the most abundant active compounds, find typical regions of elution, understand the compounds’ provenience (i.e., phenols, coming from the degradation of lignin), and to adjust the pyrolysis conditions.

## 4. Conclusions

The present study provides data on the chemical and physical characteristics of coffee silver skin. Our findings show the advantage of the recovery of CSS that can help to lower the food waste generated by coffee production. More precisely, CSS can be considered a co-product of coffee beans and become a cheap but valuable integrator of fibers, bioactive molecules, and minerals such as calcium and potassium. Different proveniences of coffee beans and compositions of coffee blends after the roasting process can cause slight differences in mineral content and phenolic profile. As shown here, common features of the composition, fibers, minerals, and phenolic species can be seen. The aim for the characterization of CSS from this study is to encourage its use in different formulations, and the acrylamide content in each specific recipe should be evaluated for the maximum allowed CSS content.

This is a starting point of a deeper study to comprehend the best conditions for the use of this by-product and how to develop new formulations in which coffee silver skin could have a positive role in the control of quality and stability, in compliance with sustainable food production.

## Figures and Tables

**Table 1:** Time and gradient (B phase, %) used for the HPLC-DAD analysis of phenolic acids.

Time (min)	B Phase (%)
0	2
20	32
30	40
40	95
45	5
52	95
55	95

**Table 2:** Physico-chemical, color, and chemical characterization of CSS.

a_w_	0.32 ± 0.004
pH	5.34 ± 0.02
L*	20.8 ± 2.62
a*	4.27 ± 0.18
b*	13.4 ± 0.80
C*	14.12 ± 0.79
Moisture (%)	2.78 ± 0.30
Ash (%)	6.79 ± 0.78
Proteins (%)	18.15 ± 2.17
Lipids (%)	2.31 ± 0.50
Fibers (%)	51.05 ± 0.35
Soluble Fibers (%)	7.86 ± 1.01
Insoluble Fibers (%)	48.85 ± 0.83
Acrylamide (μg kg^−1^)	720 ± 110
WHC (g water/g CSS)	4.89 ± 0.36
OHC (g oil/g CSS)	3.02 ± 0.11

**Table 3:** Total polyphenol content (TPC), caffeic and chlorogenic acids, caffeine content, and radical-scavenging activity (TEAC) in CSS.

Phenolic Fraction	TPC (mg GAE 100 g^−1^)	TEAC (mmol Trolox eq 100 g^−1^)	Chlorogenic Acid (mg g^−1^)	Caffeic Acid (mg g^−1^)	Caffeine (mg g^−1^)
Soluble	578 ± 64	1.57 ± 0.04	0.39 ± 0.02	0.31 ± 0.01	17.45 ± 0.69
Bound	467 ± 29	1.11 ± 0.07	0.18 ± 0.01	0.21 ± 0.01	n.d.
Sign.	**	**	**	*	-

Legend: n.d.: not detectable; asterisks indicate significance at *: *p* < 0.05 and **: *p* < 0.0.

**Table 4:** Mineral composition (mg Kg^−1^) of CSS.

Minerals	(mg Kg^−1^)
Calcium	5465 ± 343
Magnesium	2227 ± 26
Phosphorus	1462 ± 27
Potassium	14,600 ± 387
Sodium	115.85 ± 2.31
Copper	72.15 ± 4.70
Chromium	5.55 ± 0.14
Iron	212.46 ± 6.56
Manganese	23.13 ± 2.23
Nickel	3.17 ± 0.23
Zinc	18.06 ± 1.56
Aluminum	223.67 ± 16.38

**Table 5:** Qualitative profile (area %) of main volatile compounds (VOCs) from CSS.

VOCs from CSS	Area %
Aldehydes	
3-Furaldehyde	1.32
Benzaldehyde	2.51
Butanal, 2-methyl-	2.95
Butanal, 3-methyl-	3.19
Heptanal	1.51
Hexanal	8.83
Pentanal	0.81
Propanal, 2-methyl-	2.20
Alcohols	
1-Methylcyclopropanemethanol	3.00
1,3-Butanediol, (S)-	1.26
2-Furanmethanol	0.47
2-Furanmethanol, acetate	0.47
2-Nonen-1-ol	7.51
2-Octen-1-ol, (E)-	0.23
2-Octyn-1-ol	0.34
3-Hexen-1-ol, 2-ethyl-	0.43
Ketones	
2-Propanone, 1-(acetyloxy)-	1.11
Nitrogen-containing species	
(2-Aziridinylethyl)amine	1.30
3-Amino-2-oxazolidinone	1.98
Carane, 4,5-epoxy-, trans	1.58
Hydroxyurea	0.72
O-Methylisourea	0.43
Propanamide, 2-hydroxy-	0.32
Pyrazines	
1-Methyl-3-Phenylpiperazine	0.69
Pyrazine, methyl-	4.38
Pyrazine, 2-ethyl-6-methyl-	2.40
Pyrazine, 2-ethyl-3-methyl-	6.65
Pyrazine, 3-ethyl-2,5-dimethyl-	1.75
Pyrazole, 3,5-dimethyl-1-allyl-	0.78
Hydrocarbons	
Ethylbenzene	1.08
Pentane, 1-(2-propenyloxy)-	1.44
Undecane,1,2-dibromo -2-methyl-	1.64
Other compounds	
3(2H)-Furanone,dihydro-2 methyl-	0.85
α-Pinene	0.27
Furan, 2-pentyl-	1.13
Methyl glyoxal	2.0
Pyrimidine, 4-methyl-	0.57
Pyrimidine, 2-methyl-	15.4

## References

[1] Bahadur K.C., Dias G.M., Veeramani A., Swanton C.J., Fraser D., Steinke D., Lee E., Wittman H., Farber J.M., Dunfield K. (2018). When too much isn’t enough: Does current food production meet global nutritional needs?. PLoS ONE.

[2] Bessada S.M.F., Alves R.C., Oliveira M.B.P.P. (2018). Coffee Silverskin: A Review on Potential Cosmetic Applications. Cosmetics.

[3] Balentić J.P., Ačkar Đ., Jokić S., Jozinović A., Babić J., Miličević B., Šubarić D., Pavlovic N. (2018). Cocoa Shell: A By-Product with Great Potential for Wide Application. Molecules.

[4] Klingel T., Kremer J.I., Vera Gottstein V., Rajcic de Rezende T., Schwarz S., Lachenmeier D.W. (2020). A Review of Coffee By-Products Including Leaf, Flower, Cherry, Husk, Silver Skin, and Spent Grounds as Novel Foods within the European Union. Foods.

[5] Murthy P.S., Naidu M.M. (2012). Sustainable management of coffee industry by-products and value addition—A review. Resour. Conserv. Recycl..

[6] Jannissen B., Huynh T. (2018). Chemical composition and value-adding applications of coffee industry byproducts: A review. Resour. Conserv. Recycl..

[7] Alves R.C., Rodrigues F., Nunes M.A., Vinha A.F., Oliveira M.B.P.P. (2017). State of the art in coffee processing by-products (chapter 1). Handbook of Coffee Processing By-Products.

[8] Gobena Gemechu F. (2020). Embracing nutritional qualities, biological activities and technological properties of coffee byproducts in functional food formulation. Trends Food Sci. Technol..

[9] Costa A.S.G., Alves R.C., Vinha A.F., Costa C.S.G., Costa M.A., Nunes A.A., Almeida A., Santos-Silva Oliveira M.B.P.P. (2018). Nutritional, chemical and antioxidant/pro-oxidant profiles of silverskin, a coffee roasting by-product. Food Chem..

[10] Iriondo-DeHond A., Garcia N.A., Fernandez-Gomez B., Guisantes-Batan E., Escobar F.V., Blanch G.P., San Andres M.I., Sanchez-Fortun S., del Castillo M.D. (2019). Validation of coffee by-products as novel food ingredients. Innov. Food Sci. Emerg. Technol..

[11] Esquivel P., Jiménez V.M. (2012). Functional properties of coffee and coffee by-products. Food Res. Int..

[12] Machado S., Costa A.S., Pimentel F., Oliveira M.B., Alves R.C. (2020). A study on the protein fraction of coffee silverskin: Protein/non-protein nitrogen and free and total amino acid profiles. Food Chem..

[13] Pourfarzad A., Mahdavian-Mehr H., Sedaghat N. (2013). Coffee silverskin as a source of dietary fiber in bread-making: Optimization of chemical treatment using response surface methodology. LWT-Food Sci. Technol..

[14] Bertolino M., Barbosa-Pereira L., Ghirardello D., Botta C., Rolle L., Guglielmetti A., Borotto C., Dalla Vecchia S., Zeppa G. (2019). Coffee silverskin as nutraceutical ingredient in yogurt: Its effect on functional properties and its bioaccessibility. J. Sci. Food Agric..

[15] García-Serna E., Martinez–Saez N., Mesias M., Morales F.J., del Castillo M.D. (2014). Use of Coffee Silverskin and Stevia to Improve the Formulation of Biscuits. Pol. J. Food Nutr. Sci..

[16] Santos Ribeiro V., Leitão A.E., Cochicho Ramalho J., Cebola Lidon F. (2014). Chemical characterization and antioxidant properties of a new coffee blend with cocoa, coffee silverskin and green coffee minimally processed. Food Res. Int..

[17] Martinez-Saez N., Ullate M., Martin-Cabrejas M.A., Martorell P., Genovés S., Ramon D., del Castillo M.D. (2014). A novel antioxidant beverage for body weight control based on coffee silverskin. Food Chem..

[18] Walker J.M., Mennella I., Ferracane R., Tagliamonte S., Holik A.K., Hölz K., Somoza M.M., Somoza V., Fogliano V., Vitaglione P. (2020). Melanoidins from coffee and bread differently influence energy intake: A randomized controlled trial of food intake and gut-brain axis response. J. Funct. Food.

[19] European Union (2017). European Union Commission implementing regulation (EU) 2017/2468 of 20 December 2017 laying down administrative and scientific requirements concerning traditional foods from third countries in accordance with Regulation (EU) 2015/2283 of the European Parliament and of the Council on novel foods. Off. J. Eur. Union.

[20] European Union (2017). European Union Commission implementing regulation (EU) 2017/2469 of 20 December 2017 laying down administrative and scientific requirements for applications referred to in Article 10 of Regulation (EU) 2015/2283 of the European Parliament and of the Council on novel foods. Off. J. Eur. Union.

[21] AOAC (2012). Official Methods of Analysis of AOAC International, Methods 2009.01, and 2011.25.

[22] Folch J., Lees M., Sloane-Stanley G. (1957). A simple method for the isolation and purification of total lipids from animal tissues. J. Biol. Chem..

[23] Ballesteros L.F., Teixeira J.A., Mussatto S.I. (2014). Chemical, functional, and structural properties of spent coffee grounds and coffee silverskin. Food Bioprocess. Technol..

[24] Di Mattia C.D., Martuscelli M., Sacchetti G., Scheirlinck I., Beheydt B., Mastrocola D., Pittia P. (2013). Effect of Fermentation and Drying on Procyanidins, Antiradical Activity and Reducing Properties of Cocoa Beans. Food Bioprocess. Technol..

[25] Horžić D., Komes D., Belščak A., Kovačević Ganić K., Iveković D., Karlović D. (2009). The composition of polyphenols and methylxanthines in teas and herbal infusions. Food Chem..

[26] Rivero-Perez M., Perez-Magarino S., Gonzalez-San J. (2012). Role of melanoidins in sweet wines. Anal. Chim. Acta.

[27] Delgado-Ospina J., Di Mattia C.D., Paparella A., Mastrocola D., Martuscelli M., Chaves-Lopez C. (2020). Effect of Fermentation, Drying and Roasting on Biogenic Amines and Other Biocompounds in Colombian Criollo Cocoa Beans and Shells. Foods.

[28] UNI EN 13657:2002. Characterization of waste—Digestion for subsequent determination of aqua regia soluble portion of elements.

[29] Qi J., Wang H.H., Zhou G.H., Xu X.L., Li X., Bai Y., Yu X. (2017). Evaluation of the taste-active and volatile compounds in stewed meat from the Chinese yellow-feather chicken breed. Int. J. Food Prop..

[30] Tores de la Cruz S., Iriondo-DeHond A., Herrera T., Lopez-Tofiño Y., Galvez-Robleño C., Prodanov M., Velazquez-Escobar F., Abalo R., Del Castillo M.D. (2019). An Assessment of the Bioactivity of Coffee Silverskin Melanoidins. Foods.

[31] Mathlouthi M. (2001). Water content, water activity, water structure and the stability of foodstuffs. Food Control.

[32] Sacchetti G., Di Mattia C.D., Pittia P., Mastrocola D. (2009). Effect of roasting degree, equivalent thermal effect and coffee type on the radical scavenging activity of coffee brews and their phenolic fraction. J. Food Eng..

[33] Gocmen D., Sahan Y., Yildiz E., Coskun M., Aroufai I.A. (2019). Use of coffee silverskin to improve the functional properties of cookies. J. Food Sci Technol..

[34] Ateş G., Elmacı Y. (2018). Coffee silverskin as fat replacer in cake formulations and its effect on physical, chemical and sensory attributes of cakes. LWT.

[35] Ateş G., Elmacı Y. (2019). Physical, chemical and sensory characteristics of fiber-enriched cakes prepared with coffee silverskin as wheat flour substitution. J. Food Meas. Charact..

[36] Narita Y., Inouye K. (2014). Review on utilization and composition of coffee silverskin. Food Res. Int..

[37] Soliman G.A. (2019). Dietary Fiber, Atherosclerosis, and Cardiovascular Disease. Nutrients.

[38] Dong Y., Chen L., Gutin B., Haidong Z. (2019). Total, insoluble, and soluble dietary fiber intake and insulin resistance and blood pressure in adolescents. Eur. J. Clin. Nutr..

[39] González-Aguilar G.A., Blancas-Benítez F.J., Sáyago-Ayerdi S.G. (2017). Polyphenols associated with dietary fibers in plant foods: Molecular interactions and bioaccessibility. Curr. Opin. Food Sci..

[40] Food and Nutrition Board (2002). Dietary Reference Intakes for Energy, Carbohydrates, Fiber, Protein, and Aminoacids.

[41] Prasadi N.V.P., Joyce I.J. (2020). Dietary Fibre from Whole Grains and Their Benefits on Metabolic Health. Nutrients.

[42] Slavin J.L., Lloyd B. (2012). Health Benefits of Fruits and Vegetables American Society for Nutrition. Adv. Nutr..

[43] (2017). Commission regulation (EU) 2017/2158 of 20 November 2017 establishing mitigation measures and benchmark levels for the reduction of the presence of acrylamide in food. Off. J. Eur. Union.

[44] Bagdonaite K., Derler K., Murkovic M. (2008). Determination of acrylamide during roasting of coffee. J. Agric. Food Chem..

[45] Behrouzian F., Amini A.M., Alghooneh A., Razavi S.M.A. (2016). Characterization of dietary fiber from coffee silverskin: An optimization study using response surface methodology. Bioact. Carbohydr. Diet. Fibre.

[46] Belmiro R.H., Artigiani Lima Tribst A., Cristianini M. (2018). Application of high pressure homogenization on gums. J. Sci. Food Agric..

[47] Belmiro R.H., de Carvalho Oliveira L., Geraldi M.V., Junior M.R., Cristianini M. (2021). Modification of coffee coproducts by-products by dynamic high pressure, acetylation and hydrolysis by cellulase: A potential functional and sustainable food ingredient. Innov. Food Sci. Emerg. Technol..

[48] Ignat I., Volf I., Popa V.I. (2011). A critical review of methods for characterisation of polyphenolic compounds in fruits and vegetables. Food Chem..

[49] Çelik E.E., Gökmen V. (2021). Interactions between free and bound antioxidants under different conditions in food systems. Crit. Rev. Food Sci. Nutr..

[50] Guglielmetti A., Fernandez-Gomez B., Zeppa G., Del Castillo M.D. (2019). Nutritional quality, potential health promoting properties and sensory perception of an improved gluten-free bread formulation containing inulin, rice protein and bioactive compounds extracted from coffee byproducts. Pol. J. Food Nutr. Sci..

[51] Mesías M., Navarro M., Martínez-Saez N., Ullate M., del Castillo M.D., Morales F.J. (2014). Antiglycative and carbonyl trapping properties of the water soluble fraction of coffee silverskin. Food Res. Int..

[52] Bresciani L., Calani L., Bruni R., Brighenti F., Del Rio D. (2014). Phenolic composition, caffeine content and antioxidant capacity of coffee silverskin. Food Res. Int..

[53] Borrelli R.C., Esposito F., Napolitano A., Ritieni A., Fogliano V. (2004). Characterization of a New Potential Functional Ingredient: Coffee Silverskin. J. Agric. Food Chem..

[54] Bessada S.M.F., Alves R.C., Costa A.S.G., Nunes M.A., Oliveira M.B.P.P. (2018). Coffea canephora silverskin from different geographical origins: A comparative study. Sci. Total Environ..

[55] Mesías M., Delgado- Andrade C. (2017). Melanoidins as a potential functional food ingredient. Curr. Opin. Food Sci..

[56] Soetan K.O., Olaiya C.O., Oyewole O.E. (2010). The importance of mineral elements for humans, domestic animals and plants: A review. Afr. J. Food Sci..

[57] Hardisson A., Revert C., Gonzales-Weler D., Rubio C. (2017). Aluminium Exposure Through the Diet. Food Sci. Nutr..

[58] FAO, WHO (2001). Human Vitamin and Mineral Requirements.

[59] EFSA Panel on Dietetic Products, Nutrition and A (NDA) (2010). Scientific Opinion on principles for deriving and applying Dietary Reference Values. EFSA J..

[60] World Health Organization (2009). Guideline: Potassium Intake for Adults and Children. Geneva. http://www.who.int/nutrition/publications/guidelines/potassium_intake/en/.

[61] Sharma Ashimav D. (2013). Low Nickel Diet in Dermatology. Ind. J. Dermatol..

[62] Vītola V., Ciproviča I. (2016). The Effect of Cocoa Beans Heavy and Trace Elements on Safety and Stability of Confectionery Products. Rural Sustain. Res..

[63] (2018). European Food Safety Authority (EFSA). https://efsa.onlinelibrary.wiley.com/doi/full/10.2903/j.efsa.2018.5372.

[64] European Food Safety Authority (EFSA) (2011). On the Evaluation of a New Study Related to the Bioavailability of Aluminium in Food. EFSA J..

[65] European Food Safety Authority (EFSA) (2020). Update of the risk assessment of Nickel in food and drinking water. EFSA J..

[66] Angeloni S., Scortichini S., Fiorini D., Sagratini G., Vittori S., Neiens S.D., Steinhaus M., Zheljazkov V.D., Maggi F., Caprioli G. (2020). Characterization of Odor-Active Compounds, Polyphenols, and Fatty Acids in Coffee Silverskin. Molecules.

[67] Caporaso N., Whitworth M.B., Cui C., Fisk I.D. (2018). Variability of single bean coffee volatile compounds of Arabica and robusta roasted coffees analysed by SPME-GC-MS. Food Res. Int..

[68] De Toledo P.A.B., de Melo M.M.R., Pezza H.R., Toci A.T., Pezza L., Silva C.M. (2017). Discriminant analysis for unveiling the origin of roasted coffee samples: A tool for quality control of coffee related products. Food Control.

[69] Yang N., Liu C., Liu X., Kreuzfeldt Degn T., Munchow M., Fisk I. (2016). Determination of volatile marker compounds of common coffee roast defects. Food Chem..

[70] Fisk I.D., Kettle A., Hofmeister S., Virdie A., Kenny J.S. (2012). Discrimination of roast and ground coffee aroma. Flavour.

[71] Dos Santos Polidoro A., Scapin E., Lazzari E., Nunes Silva A., Loreiro dos Santos A., Bastos Caramão E., Assis Jacques R. (2018). Valorization of coffee silverskin industrial waste by pyrolysis: From optimization of bio-oil production to chemical characterization by GC × GC/qMS. J. Anal. Appl. Pyrol..

